# Multifunctional acyltransferase HBO1: a key regulatory factor for cellular functions

**DOI:** 10.1186/s11658-024-00661-y

**Published:** 2024-11-14

**Authors:** Zhanhuan Su, Yang Zhang, Jingqiong Tang, Yanhong Zhou, Chen Long

**Affiliations:** 1 Department of General Surgery, The Second Xiangya Hospital of Central South University, Changsha, 410011 Hunan China; 2 Cancer Research Institute, Basic School of Medicine, Central South University, Changsha, 410078 Hunan China; 3 Department of Geriatrics, The Second Xiangya Hospital of Central South University, Changsha, 410011 Hunan China

**Keywords:** HBO1, Acetylation, Ubiquitylation, DNA replication, Immune regulation

## Abstract

HBO1, also known as KAT7 or MYST2, is a crucial histone acetyltransferase with diverse cellular functions. It typically forms complexes with protein subunits or cofactors such as MEAF6, ING4, or ING5, and JADE1/2/3 or BRPF1/2/3, where the BRPF or JADE proteins serve as the scaffold targeting histone H3 or H4, respectively. The histone acetylation mediated by HBO1 plays significant roles in DNA replication and gene expression regulation. Additionally, HBO1 catalyzes the modification of proteins through acylation with propionyl, butyryl, crotonyl, benzoyl, and acetoacetyl groups. HBO1 undergoes ubiquitination and degradation by two types of ubiquitin complexes and can also act as an E3 ubiquitin ligase for the estrogen receptor α (ERα). Moreover, HBO1 participates in the expansion of medullary thymic epithelial cells (mTECs) and regulates the expression of peripheral tissue genes (PTGs) mediated by autoimmune regulator (AIRE), thus inducing immune tolerance. Furthermore, HBO1 influences the renewal of hematopoietic stem cells and the development of neural stem cells significantly. Importantly, the overexpression of HBO1 in various cancers suggests its carcinogenic role and potential as a therapeutic target. This review summarizes recent advancements in understanding HBO1’s involvement in acylation modification, DNA replication, ubiquitination, immunity, and stem cell renewal.

## Introduction

HBO1, also known as KAT7 or MYST2, belongs to the MYST acetyltransferase family and is primarily responsible for histone H3 and H4 acetylation [1–4]. It exerts diverse roles in crucial cellular processes, including DNA replication and repair, gene transcription, protein ubiquitination, immune regulation, stem cell pluripotency and self-renewal maintenance, and embryonic development. Initially identified as an interaction partner for the largest subunit ORC1 of the Origin Recognition Complex (ORC) [4], HBO1 is a 611-amino-acid protein comprising a unique N-terminal serine-rich region (22% in aa 1–167) and a conserved C-terminal domain of 270 amino acids specific to the MYST protein family. The MYST domain encompasses an acetyl-CoA binding region, facilitating histone acetylation, and an atypical C2HC zinc finger [5]. Studies suggest that the N-terminal domain (NTD) of HBO1 harbors a transcriptional inhibitory domain, capable of downregulating androgen receptor expression and sequestering an essential cofactor from the NF-κB transcription complex, thereby reducing NF-κB activity [6, 7]. However, the crystal structure of NTD remains largely unexplored. Intriguingly, HBO1 (311–611) displays higher activity than HBO1 (1–611) in vitro, implying a potential negative regulation of the histone acetyltransferase (HAT) activity of the C-terminal MYST domain by the NTD of HBO1 [8]. Therefore, protein modifications such as phosphorylation or cofactor/substrate binding may serve as pathways to alleviate this negative regulation and fully unleash the HAT activity of HBO1.

Histone acetylation stands as a central function of HBO1. Acetylation, a prevalent protein modification in cells, holds pivotal roles in diverse cellular processes including cell proliferation, gene transcription, and signal transduction. Specifically, in the context of genomic histones, acetylation induces structural relaxation, thereby exposing DNA sites for crucial processes such as replication and transcription [9, 10]. The addition or removal of acetyl groups from histone lysine residues is mediated by lysine acetyltransferases (KATs) and deacetyltransferases, respectively. As a member of the KAT family, the functional mechanism and specific impact of HBO1 are still not fully elucidated. Moreover, the observed high expression of HBO1 in various cancer cells suggests its potential as a target for cancer treatment [11]. In summary, our understanding of HBO1 remains incomplete, underscoring its substantial research value and warranting further investigation.

This article presents a comprehensive overview of HBO1 as a multifunctional acyltransferase, which is a key factor of cell functions, emphasizing its regulatory roles in DNA replication and participation in DNA repair. Additionally, HBO1’s involvement in ubiquitination, where it can be ubiquitinated itself and also acts as a ubiquitin ligase, is explored. The crucial role of HBO1 in immune regulation and T-cell development is highlighted, alongside its contribution to the regulation of stem cell pluripotency and self-renewal. Moreover, the article delves into the association between HBO1 and various diseases, including malignant tumors and chronic obstructive pulmonary disease, with the aim of providing a fresh perspective for a comprehensive and systematic understanding of the multifaceted functions and mechanisms of the acyltransferase HBO1.

## HBO1 is a multifunctional acyltransferase

As a member of the histone acetyltransferase family, HBO1 typically forms protein complexes with various cofactors or partner proteins, serving as the core catalytic subunit to exert its function. The identified HBO1 protein complex primarily consists of HBO1, ING4/5, MEAF6, and BRPF1/2/3 or JADE1/2/3 [12]. ING4 and ING5, belonging to the ING tumor suppressor family, regulate the cell cycle and apoptosis [13, 14]. H3K4me3 is a mark that is found near the transcription start site (TSS) of actively transcribed genes [15]. The N-terminal domain of ING4/5 forms homodimers or heterodimers, recognizing the histone H3 lysine 4 trimethylation (H3K4me3) site through the C-terminal PHD domain, subsequently recruiting the HBO1 complex to promote histone acetylation [13, 14, 16–19]. BRPF or JADE serve as scaffold proteins for the HBO1 complex, enhancing its acetylation function [2, 16, 20, 21]. BRPF primarily targets histone H3, while JADE predominantly targets H4 [3, 17]. The BRPF protein typically comprises an N-terminal PHD-C2H2 zinc finger-PHD domain (PZP), a central bromodomain, and a C-terminal PWWP domain [17, 22]. When HBO1 binds to BRPF1, the complex’s action on chromatin acetylation is confined to histone H3, resulting in the increases of H3K23ac and H3K14ac [17]. The interaction between BRPF2 and HBO1 is primarily localized to a short N-terminal region of the former and the MYST domain of the latter. The simultaneous binding of both to histone proteins allows for the correct positioning of the N-terminal tails of the histones at the acetyltransferase active site of HBO1, thereby enhancing the acetyltransferase activity of HBO1. The binding of the N-terminal region of BRPF2 may also stabilize HBO1 in a more physiological conformation, thereby enhancing its interaction with histone substrates, ultimately boosting the acetyltransferase activity of HBO1 [21]. Additionally, compared with HBO1 alone, the BRPF3 complex enhances the levels of H3K9ac, H3K14ac, and H4K16ac [22]. JADE-1 is the most prevalent member of the JADE family and serves as the essential cofactor for the HBO1 acetyltransferase complex [23]. Similar to BRPF, JADE also possesses two PHD domains, capable of synergistic action with ING4/5 to enhance HBO1-mediated histone acetylation [2, 14, 16]. Additionally, phosphorylation of JADE1 during the cell cycle may regulate the removal of HBO1 complexes from chromatin, facilitating histone deacetylation during mitosis [24].

Remarkably, these subunits are not exclusive to the HBO1 complex, and some subunits are involved in the composition of other HAT complexes. Studies have shown that JADE1 physically associates to Tip60 HAT and enhances the acetylation targeting H4 [25]. In addition, BRPF, ING5, and MEAF6 are involved in the composition of MOZ/MORF complex. Similar to its role in HBO1 complex, BRPF acts as a scaffold in MOZ/MORF complex to connect MOZ/MORF with ING5 and MEAF6. Additionally, BRPF is also important for activating the MOZ/MORF complex. Just like its function in HBO1 complex, BRPF is able to upregulate the acetyltransferase activity of MOZ/MORF [26]. ING5 targets H3K4me3 at the promoter of active transcription region and recruits the MOZ/MORF complex for histone acetylation [27]. In conclusion, by studying these subunits shared between the HBO1 complex and other HAT complexes, we have a clearer understanding that their roles in the HBO1 complex are universal.

**Fig. 1:**
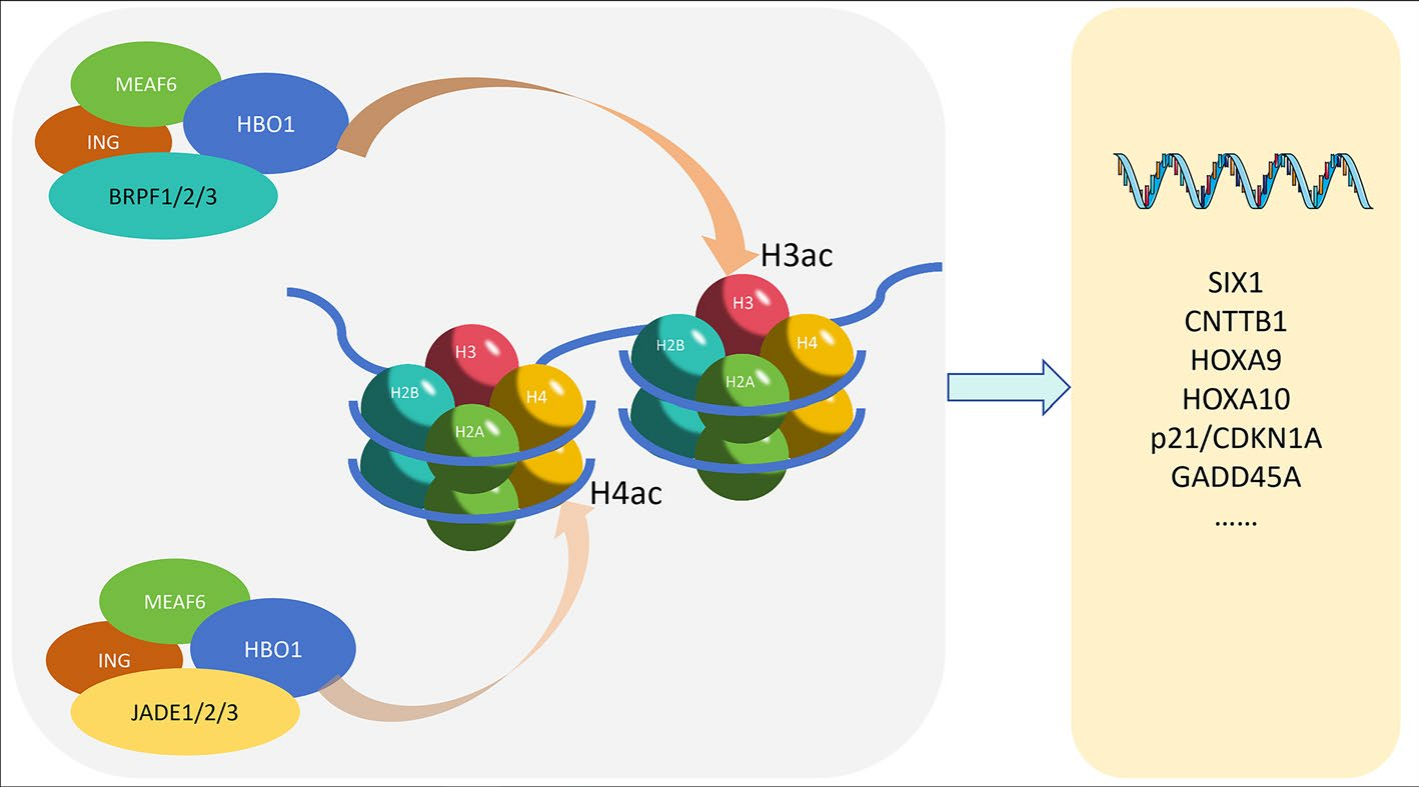
HBO1 complexes and gene expression. HBO1 forms complexes with other proteins to acetylate histones and promote the expression of multiple genes. The HBO1 complex with BRPF as the scaffold mainly targets histone H3 acetylation, while the HBO1 complex with JADE as the scaffold mainly targets histone H4 acetylation. The histone acetylation will promote the expression of many genes including *SIX1*, *CNTTB1*, *HOXA9*, *HOXA10*, *p21/CDKN1A*, and *GADD45A*

HBO1 significantly influences gene expression, signal transduction, and cellular growth by affecting acetylation function. HBO1 is located at the transcriptional start site (TSS) of active genes, where it directly acetylates histones, thereby regulating gene transcription. The strength of acetylation signal HBO1 signaling correlates closely with gene expression levels [14, 28]. For instance, HBO1 is recruited to the promoter of glycolysis-related genes by the transcription factor SIX1, facilitating their transcription through H4K5 acetylation. *HBO1* knockdown or knockout leads to decreased glucose uptake, pyruvate levels, lactate production, adenosine triphosphate (ATP) levels, and extracellular acidification rate (ECAR), while oxygen consumption rate (OCR) increases. In cancer cells, SIX1 enhances the Warburg effect via this pathway [29]. Additionally, HBO1 promotes *CTNNB1* gene transcription by acetylating H3K14, H4K8, and H4K12, thereby activating the Wnt/β-catenin signaling pathway [30]. In leukemia, HBO1 upregulates *HOXA9* and *HOXA10* expression through H3K14 acetylation, maintaining leukemia stem cell characteristics [31, 32]. Moreover, the NUP98–HBO1 fusion protein induces abnormal histone acetylation, leading to increased acetylation levels at the *HOXA9* promoter on H4K8, H4K12, and H3K14 and activation of carcinogenic features in chronic myeloid mononuclear leukemia (CMML) [33]. HBO1 also participates in p53-mediated transcriptional activation of *p21/CDKN1A* and *GADD45A* through its HAT active site [34]( Fig. 1).

However, recent research on BRPF2–HBO1 and JADE1–HBO1 complexes has challenged the traditional view of HBO1’s substrate specificity. It has been found that HBO1 is not restricted to histone H3 and H4 acetylation, as previously believed. In addition to acetylation, HBO1 can catalyze propionylation, butyrylation, and crotonylation both in vivo and in vitro. Furthermore, HBO1 can extend its catalytic activity to histone H2. Interestingly, the specific targeting function of BRPF and JADE appears to be less distinct, leading to considerable overlap in catalytic sites on histones H3 and H4 within the HBO1 complex [28]. This intriguing observation warrants further investigation to fully understand its implications. Additionally, lysine benzoylation (Kbz) has emerged as a novel posttranslational modification involved in chromatin remodeling, transcriptional regulation, and tumor growth. HBO1 has been identified as a participant in Kbz in mammals, further expanding its functional repertoire [35]. Lastly, HBO1 is also involved in histone acetyl-acetylation (Kacac) processes, adding another layer of complexity to its regulatory mechanisms [36].

In summary, HBO1 emerges as a multifunctional acyltransferase with significant involvement in histone acetylation and potentially nonhistone substrates. However, the interaction mechanism between HBO1 and the MEAF6 protein within the HBO1 complex remains unexplored, along with its biological implications. Furthermore, the distinctions among the BRPF1/2/3 and JADE1/2/3 families have not been systematically elucidated. It remains unclear whether their anchoring sites on histones, as scaffold proteins, exhibit consistency across these families. These unresolved questions underscore the need for further research in this field.

## HBO1 has a regulatory effect on DNA replication and is involved in DNA repair after ultraviolet irradiation

Eukaryotic DNA replication is a continuous process encompassing various tightly regulated steps, including replication origin recognition, pre-replication complex loading, and replication fork initiation. Key factors such as ORC1, CDT1, CDC6, and MCM are essential for orchestrating these steps [37]. HBO1 is widely recognized for its indispensable role in DNA replication, as evidenced by numerous studies.

**Fig. 2:**
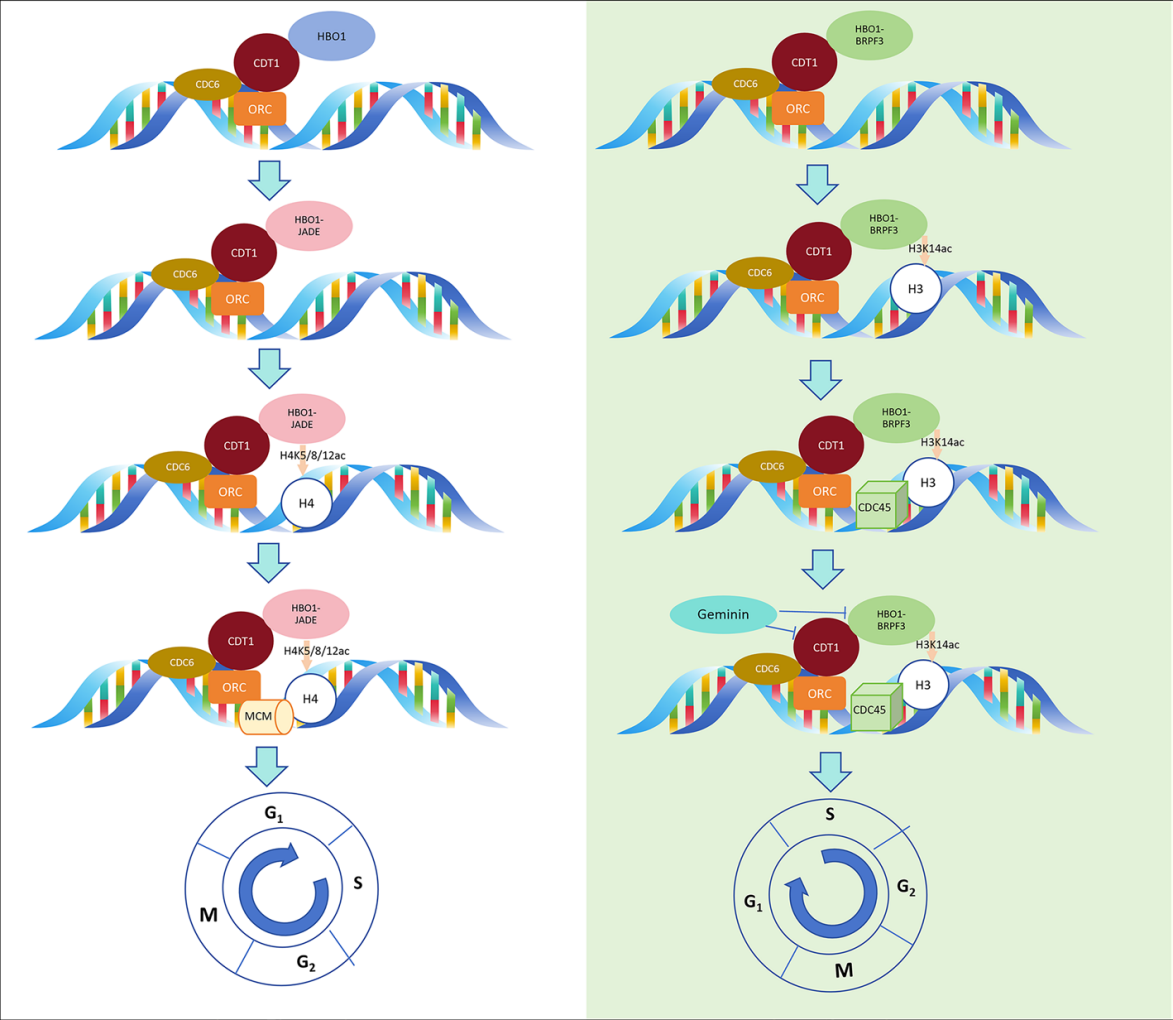
HBO1-mediated histone acetylation in DNA replication. HBO1-mediated histone acetylation promotes G1 phase DNA replication licensing and S phase DNA replication activation. In the G1 phase, the HBO1–JADE complex promotes the acetylation of H4K5/8/12 to relax the chromatin conformation, and facilitates the loading of the MCM complex to the replication starting point, promoting the assembly of the pre-replication complex. In S phase, the HBO1–BRPF3 complex specifically directs H3K14ac, thereby promoting CDC45 loading to activate S-phase DNA replication. Additionally, the regulatory protein Geminin prevents the second round of DNA replication by inhibiting the basic replication factor CDT1, possibly by inhibiting HBO1

During the assembly of the pre-replication complex (pre-RC), ORC initially recruits CDC6 and CDT1 to the replication origin in the early stage of the G1 phase [38]. Subsequently, CDT1 directly interacts with HBO1, facilitating the recruitment of HBO1 to the replication origin [39]. The HBO1–JADE complex promotes H4K5/8/12 acetylation, leading to the relaxation of chromatin conformation. This process facilitates the loading of the MCM complex onto replication origins and promotes the assembly of pre-replication complexes [39, 40]. Depletion of *Xenopus laevis* HBO1 in *Xenopus laevis* egg extracts results in the loss of MCM2-7 chromatin binding and elimination of DNA replication, indicating the necessity of HBO1 for MCM2-7 complex chromatin binding during the G1 phase, a critical step in DNA replication licensing [41]. Studies have demonstrated that HBO1 and MCM2 functions rely on the N-terminal domain of MCM2 and the C2HC zinc finger of HBO1 [5]. HBO1 directly interacts with CDT1 and enhances CDT1-dependent replication, although it is not indispensable for CDT1’s association with replication origins [39]. In the context of stress response, phosphorylation of CDT1 can inhibit the recruitment of HBO1 histone acetylase, consequently blocking replication licensing [42]. Moreover, the BRPF3 scaffold specifically guides HBO1 to H3K14ac, promoting the loading of CDC45 to activate DNA replication in the S phase [1]. Thus, through collaboration with different scaffolds, HBO1-mediated chromatin acetylation facilitates two consecutive steps in replication initiation: licensing and activation. In the S phase, the regulatory protein Geminin prevents the second round of DNA replication by inhibiting the essential replication factor CDT1. Notably, HBO1 may be inhibited to affect this process, because Geminin does not inhibit MCM loading through simple spatial interference of the CDT1–MCM2-7 interaction, but plays a role through nonspatial mechanisms [40, 43, 44]. HBO1 has been shown to acetylate ORC2, MCM2, CDC6, and Geminin in vitro, indicating its potential role in regulating the initiation of DNA replication by acetylating these factors [41]( Fig. 2).

The role of HBO1 in the origin of replication is also subject to regulation by other factors. For instance, FAD24 (adipocyte differentiation factor 24) has been identified to interact with HBO1 during the process of pre-adipocytes transitioning into adipocytes through mitotic clonal expansion (MCE). FAD24 co-localizes with HBO1 in chromatin during pre-replication complex assembly. Inhibition of FAD24 expression during adipocyte differentiation leads to reduced recruitment of HBO1 to the origin of DNA replication, while knockout of the *HBO1* gene inhibits MCE and adipogenesis. These findings suggest that FAD24 acts as an auxiliary factor in recruiting HBO1 to the origin of DNA replication [45–47]. Furthermore, under conditions of hyperosmotic stress, HBO1 can directly bind to p53, thereby inhibiting HBO1-HAT activity and subsequently impeding the loading of the MCM2-7 complex. This results in the stalling of pre-replication complex assembly. Treatment with hydroxyurea (HU), which blocks DNA replication fork progression, leads to downregulation of p53-dependent HBO1 activity [8, 11]. Interestingly, after the activity of HBO1-HAT decreased, the MCM2-7 complex still binds to chromatin. One possible explanation is that HBO1 may have other functions in the S phase, although the specific mechanisms remain unclear.

The experiments indicating that “HBO1 plays an important role in DNA replication and cell proliferation” have predominantly utilized human tumor cell lines or other immortalized cells, such as HeLa cells, C33A cells, MCF7 cells, Saos2 cells, A549 cells, and 293 T cells. Interestingly, experiments conducted using mouse embryos revealed that fibroblasts lacking HBO1, isolated from embryos, can proliferate normally and exhibit normal MCM localization. Moreover, embryos lacking HBO1 can progress through normal development up to the gastrulation stage, with developmental abnormalities and mortality occurring thereafter. Specifically, organs such as blood vessels and mesenchyme fail to differentiate and develop normally in embryos lacking HBO1, leading to a significant decrease in total RNA extraction from cells. Notably, crucial regulatory genes such as Notch1 during gastrulation development were undetected in HBO1-deficient embryos. These findings suggest that embryonic death may be attributed to inadequate gene expression products rather than defects in cell proliferation. It suggests that HBO1’s primary role in embryonic development appears to be as a gene expression activator rather than a participant in DNA replication and cell proliferation [48]. Subsequent experiments conducted by the same research team using immortalized cells such as HeLa and 293T cells revealed that loss of HBO1 had minimal impact on cell proliferation and DNA replication. Instead, loss of HBO1 primarily affected genes involved in cell adhesion, resulting in reduced cell adhesion, particularly in 293T cells, with relatively minor effects on other cellular processes [49]. Additionally, research has demonstrated that HBO1 plays a specific role at the centromere, where it interacts with M18BP1 to positively regulate the assembly of CENP-A and counteract heterochromatin-mediated centromere inactivation [50]. In summary, there remains significant scope for research and clarification regarding HBO1’s role in cell proliferation and DNA replication, as well as its overall impact. Questions persist regarding whether HBO1’s function is truly cell-intrinsic and the underlying mechanism of its identification, prompting deeper inquiry into its biological significance.

**Fig. 3:**
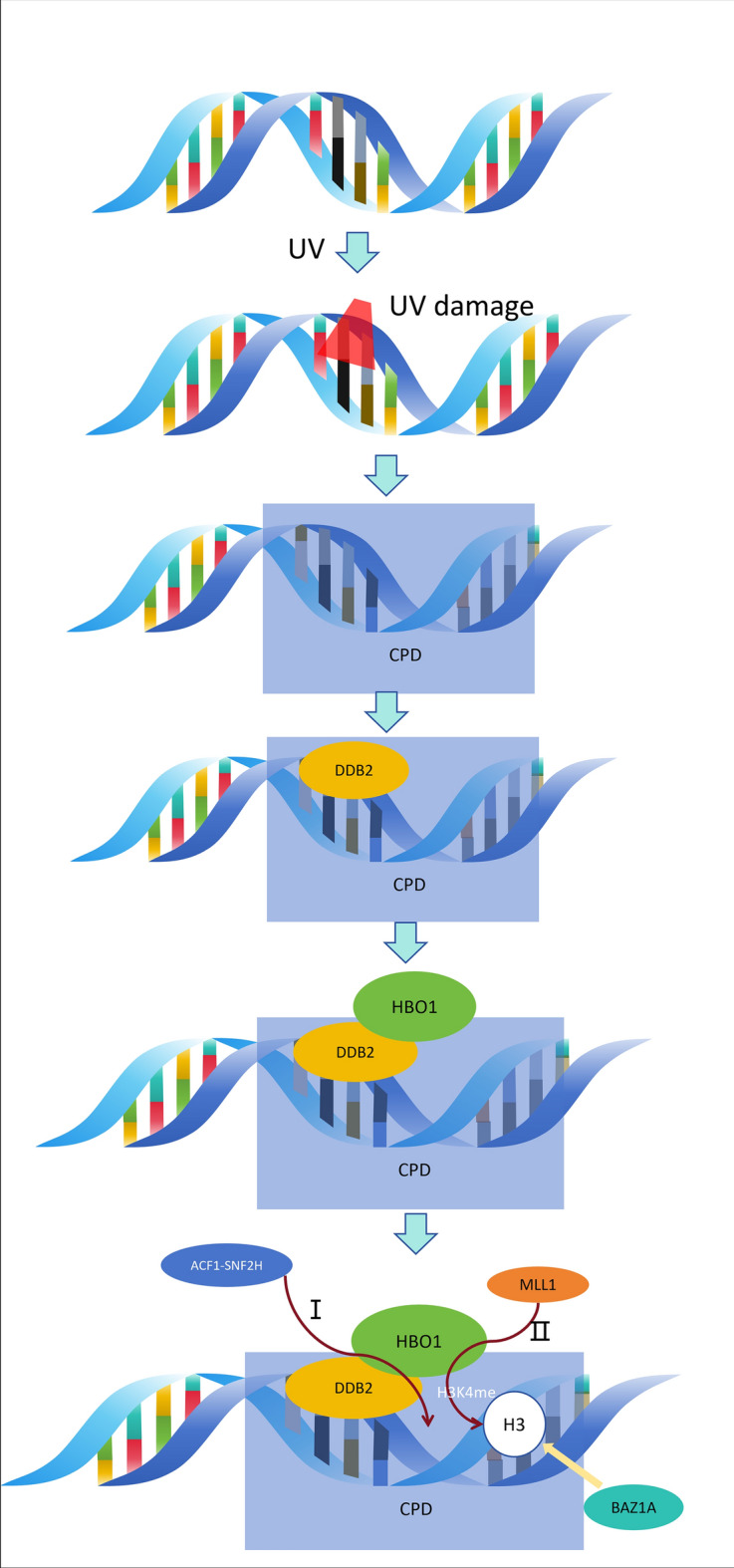
HBO1 involvement in DNA repair after UV irradiation. HBO1 plays a crucial role in the DNA repair process after UV irradiation, primarily associated with global genome nucleotide excision repair (GG-NER). Following UV-induced DNA damage, DDB2 recognizes the cyclobutyl pyrimidine dimer (CPD) site, and phosphorylated HBO1 binds to DDB2, mediating histone acetylation. HBO1 also maintains the chromatin remodeling agent ACF1–SNF2H at the damage site, inducing chromatin remodeling. Additionally, the methyltransferase MLL1 interacts with HBO1 and localizes at the UV damage site to methylate histone H3K4. BAZ1A, a subunit of the SWI/SNF chromatin remodeling factor, targets trimethylated histone H3K4 (H3K4me3). These mechanisms collectively disrupt the interaction between DNA and histones, facilitating the loading of NER factors including XPC for DNA repair

HBO1 participates in DNA repair following ultraviolet irradiation, primarily through its involvement in global genome nucleotide excision repair (GG-NER) [51]. Upon UV-induced DNA damage, the protein DDB2 recognizes sites of cyclobutyl purine dimers (CPD) and swiftly localizes to the damaged site [52, 53]. However, the densely packed chromatin structure poses a barrier to the entry of repair proteins. Therefore, histone modifications, such as acetylation and ATP-dependent chromatin remodeling, are crucial during NER to overcome these structural obstacles [54]. HBO1 plays a key role in this process by phosphorylation at Ser50 and Ser53 by ATM/ARM, facilitating its binding to DDB2 and subsequent histone acetylation [51]. Additionally, HBO1 interacts with chromatin remodeling proteins ACF1 and SNF2H [55, 56], aiding in the maintenance of ACF1–SNF2H at the damage site to induce chromatin remodeling [51]. Furthermore, HBO1 mediates the phosphorylation of methyltransferase MLL1 at Ser516, leading to its localization at UV damage sites and subsequent methylation of histone H3K4 [57]. BAZ1A, a subunit of the chromatin remodeling factor ISWI family, targets trimethylated histone H3K4 (H3K4me3), disrupting the interaction between DNA and histones and facilitating the recruitment of NER factors, including XPC, for DNA repair [51, 58, 59]. This coordinated action underscores the critical role of HBO1 in orchestrating chromatin modifications essential for efficient GG-NER (Fig. 3).

## HBO1 can be ubiquitinated and also act as a ubiquitin ligase

**Fig. 4:**
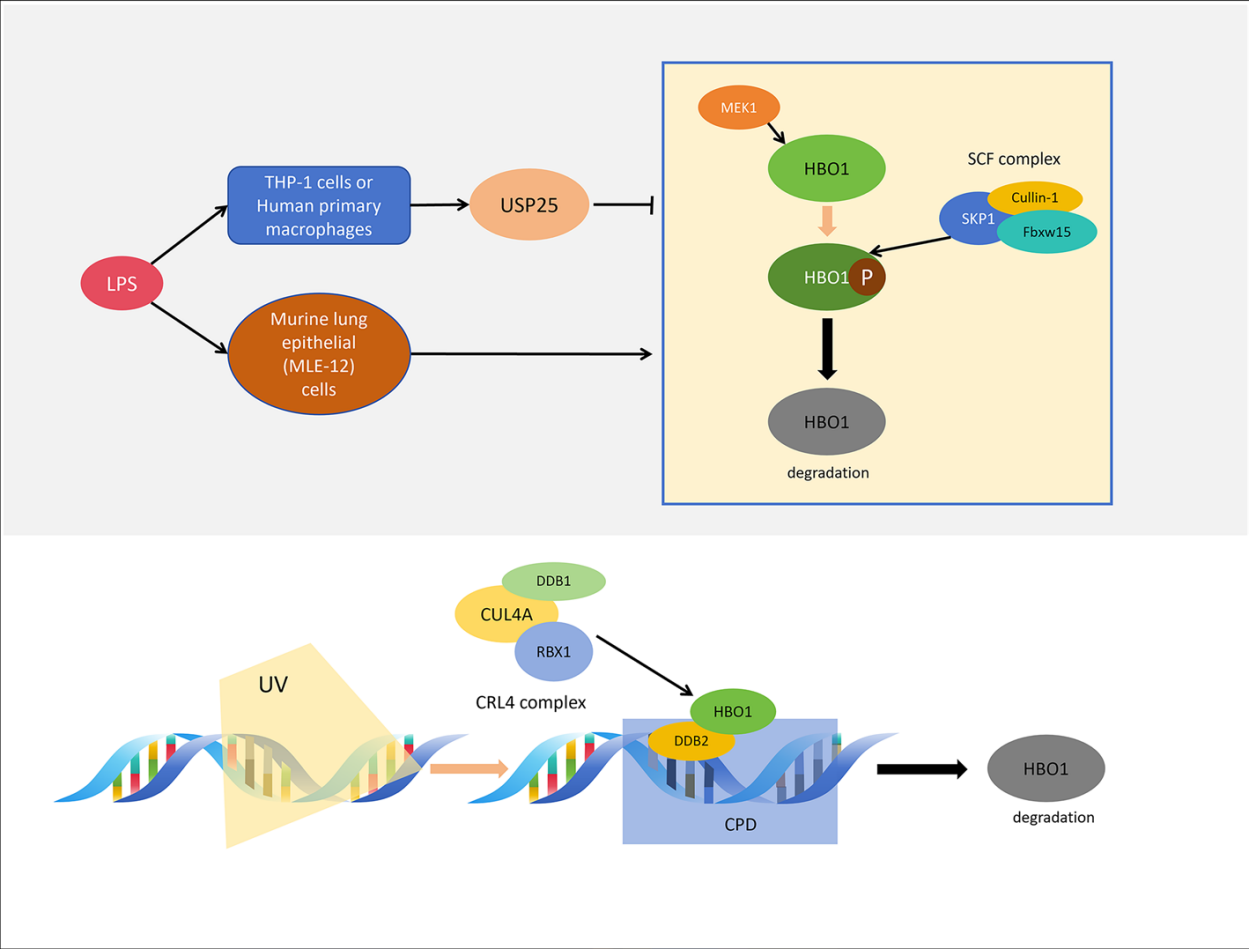
Ubiquitination and degradation of HBO1. HBO1 can undergo ubiquitination by the SCF (SKP1/Cullin-1/Fbxw15) and CRL4 (DDB1-CUL4A-RBX1) complexes. Protein kinase MEK1 phosphorylates HBO1, promoting its degradation via the Fbxw15-mediated ubiquitin proteasome pathway. In mouse lung epithelial cells (MLE-12), LPS induces HBO1 degradation through this pathway. Conversely, LPS stimulation in THP-1 monocytes and human primary macrophages inhibits HBO1 ubiquitination by increasing the level of deubiquitinase USP25 protein, resulting in varying degrees of HBO1 protein elevation. Furthermore, DNA damage caused by UV irradiation leads to the degradation of HBO1 by the DDB2-mediated CRL4 (DDB1–CUL4A–RBX1) complex

Two ubiquitin complexes, SCF (SKP1/Cullin-1/Fbxw15) and CRL4 (DDB1-CUL4A-RBX1), are involved in the ubiquitination of HBO1. Fbxw15 interacts with HBO1 to mediate its ubiquitination, leading to degradation primarily in the cytoplasm despite the presence of histone acetyltransferase activity associated with HBO1 in the nucleus. Lys338 has been identified as the receptor site for SCF-mediated ubiquitination of HBO1. Mitogen-activated protein kinase (MEK1) phosphorylates HBO1, promoting its degradation via the Fbxw15-mediated ubiquitin proteasome pathway. Studies have shown that overexpression of *MEK1* increases HBO1 degradation, while *MEK1* silencing stabilizes HBO1 acetyltransferase. Knockout of *Fbxw15* abolishes MEK1-induced HBO1 degradation, indicating Fbxw15 dependence in this process. Furthermore, *MEK1* knockout disrupts the interaction between HBO1 and Cullin1/Fbxw15 and reduces HBO1 ubiquitination in cells, suggesting MEK1’s role in HBO1 phosphorylation and subsequent degradation by Fbxw15-mediated ubiquitination. In mouse lung epithelial cells (MLE-12), endotoxin lipopolysaccharide (LPS) induces HBO1 degradation via MEK1 phosphorylation and the Fbxw15-mediated ubiquitin proteasome pathway, resulting in reduced H3K14ac levels and cell proliferation [60]. Conversely, LPS stimulation in THP-1 monocytes and human primary macrophages leads to increased HBO1 protein levels owing to elevated deubiquitinase USP25 levels, promoting HBO1 deubiquitination and stabilization. USP25-mediated deubiquitination enhances HBO1’s response to the endotoxin-induced inflammatory response, thereby boosting the transcription of interleukin (IL)-1β, IL-6, and IL-10 mediated by HBO1 [61]. Moreover, after UV irradiation-induced DNA damage, HBO1 is degraded by the DDB2-mediated CRL4 complex. Ser50 and Ser53 phosphorylation of HBO1 in an ATM/ATR-dependent manner facilitates its preferential ubiquitination by CRL4^DDB2^, essential for appropriate cell cycle arrest to complete DNA repair. Mutating Ser50 and Ser53 inhibits HBO1 phosphorylation, leading to failure in repairing DNA damage post-UV irradiation and inhibiting cell proliferation [62] (Fig. 4).

Indeed, HBO1 exhibits an intriguing dual role as an E3 ubiquitin ligase, targeting not only itself but also other proteins. In breast cancer, HBO1 functions as an E3 ubiquitin ligase to negatively regulate the stability of estrogen receptor α (ERα) [63]. Its MYST domain possesses E3 ligase activity, facilitating the proteasome-dependent degradation of ERα. Interestingly, estradiol-17β can inhibit HBO1’s E3 ligase activity on ERα in vitro, thereby attenuating ERα ubiquitination, whereas highly active ERα mutants are more susceptible to HBO1’s E3 ligase activity [64]. Whether HBO1 can exert its ubiquitin ligase function on additional proteins remains a topic worthy of further investigation.

## HBO1 is essential for immune regulation and T cell development

Thymic epithelial cells (TECs) govern the differentiation and selection of thymic T lymphocytes [65], with medullary thymic epithelial cells (mTECs) being particularly influential in negative selection of autoreactive thymocytes and the differentiation of regulatory T cells (Tregs) [66]. The autoimmune regulator (AIRE) orchestrates the transcription of numerous peripheral tissue genes (PTGs) in mTECs [66, 67]. Deficiencies in AIRE, observed in both humans and mice, lead to impaired expression of relevant PTGs in mTECs, resulting in the escape of autoreactive T cells from the thymus or the failure to induce Treg cells, thereby fostering impaired thymic negative selection and the development of multiple organ autoimmune diseases [68–70]. Recent research underscores the pivotal role of HBO1 in thymus development and the mediation of immune tolerance. Notably, highly transcribed HBO1 and abundant H3K14ac are evident across all TEC subsets. HBO1-deficient mice exhibit hypoplastic thymuses in young adulthood, characterized by significantly reduced numbers of thymic epithelial cells, particularly in mTECs. Flow cytometry analyses reveal a diminished proportion and count of AIRE^+^ cells, suggesting that HBO1 in TECs is crucial for AIRE^+^ and thymic medulla expansion. Moreover, in mice lacking HBO1, the expression of AIRE-dependent PTGs, including AIRE-induced lung antigen BPIFB9A essential for lung immune tolerance, substantially decreases compared with control groups [71, 72], while AIRE-independent PTGs remain mostly unaffected. Using the small molecule inhibitor WM-3835, which targets HBO1 function, or employing the *HBO1* gene deletion system, acute inhibition of HBO1 activity demonstrates no interference with AIRE expression, nor does it alter the AIRE protein level in TECs. However, it does impede the transcriptional activation of AIRE target genes. Mechanistically, HBO1 may enhance chromatin accessibility around the promoter of AIRE-regulated genes through its HAT activity, thereby facilitating the normal transcription of PTGs. In summary, HBO1 plays a critical role in promoting the expansion of mTECs and serves as a major regulator in AIRE-mediated PTG expression, consequently contributing to the induction of immune tolerance [73].

**Fig. 5:**
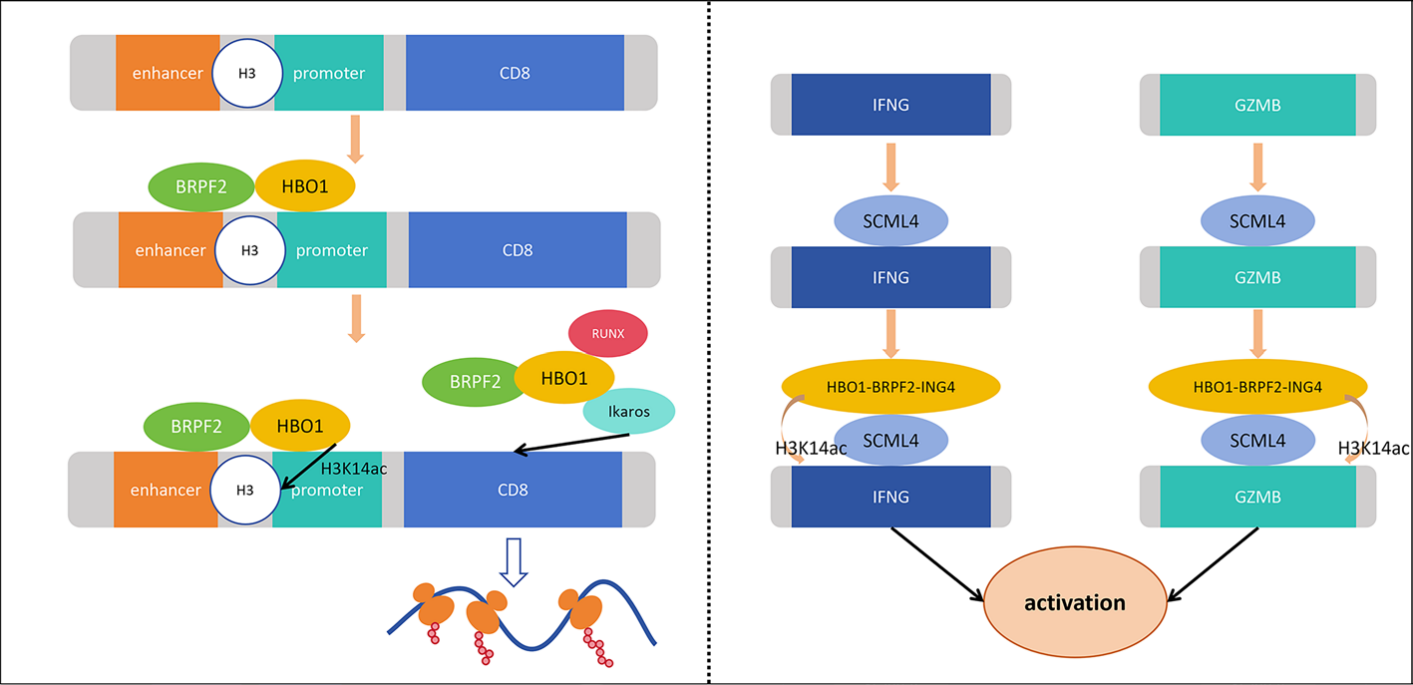
HBO1 promotes immune-related gene expression via histone acetylation. BRPF2 and HBO1 form complexes that bind to the enhancer and promoter of the *CD8* gene, respectively. Together, they acetylate histone H3 at lysine 14 (H3K14ac) at the *CD8* locus, leading to full activation of the *CD8* gene. The BRPF2–HBO1 complex also interacts directly with key regulatory factors, such as the RUNX family transcription factors and Ikaros, to activate *CD8* gene expression. SCML4, a transcription factor crucial for CD8+ resident memory T cells (Trm) and tumor-infiltrating lymphocytes (TIL), recruits the HBO1–BRPF2–ING4 complex to mediate H3K14ac, thereby enhancing chromatin accessibility during T cell activation and increasing the expression of T cell effector molecules, such as interferon-gamma (IFNG) and granzyme B (GZMB)

During T cell development, the expression of *CD8* genes undergoes regulation through the concerted action of at least five different *CD8* enhancers [74]. Recent investigations have highlighted the involvement of BRPF2 and HBO1 in this regulatory process. Specifically, BRPF2 and HBO1 form a complex responsible for H3K14 acetylation of the *CD8* locus, with BRPF2 binding to the enhancer and HBO1 binding to the promoter of the *CD8* gene. Microarray analysis and other findings have underscored the critical role of the BRPF2–HBO1 complex in acetylating H3K14 on the transcriptional regulatory elements of the *CD8* gene, a process necessary for the effective activation of the *CD8* gene. Moreover, the BRPF2–HBO1 complex has been shown to directly interact with key regulatory factors involved in *CD8* gene activation, such as RUNX family transcription factors and Ikaros [75, 76]. This complex may facilitate chromatin relaxation through H3K14ac, subsequently recruiting transcription complexes to the *CD8* enhancer to fully activate the *CD8* locus. Importantly, these findings highlight the role of the BRPF2–HBO1 complex in activating *CD8* expression rather than merely maintaining its expression [77]. Furthermore, HBO1 has been implicated in regulating the functional activity of CD8^+^ tissue-resident memory T cells (Trm) and tumor-infiltrating lymphocytes (TIL). SCML4, a transcription factor critical for Trm and TIL survival and activation, has been found to bind to components in the HBO1–BRPF2–ING4 complex through its C-terminal domain. Treatment with a BRPF2 inhibitor (NI-57) significantly reduced the expression of intracellular T cell effector molecules (IFNG and GZMB) in Jurkat cells, while treatment with an H3K14ac deacetylase inhibitor (HDAC-IN-38) notably increased their expression. Mechanistically, SCML4 recruits the HBO1–BRPF2–ING4 complex to mediate H3K14ac, thereby enhancing chromatin accessibility during T cell activation and increasing the expression of relevant genes associated with T cell function [78](Fig. 5).

## HBO1 is involved in regulating pluripotency and self-renewal of stem cells

HBO1 is involved in regulating the self-renewal of hematopoietic stem cells. Adult hematopoiesis is a tightly regulated process [79], and HBO1 has emerged as a crucial factor in maintaining hematopoietic stem cells (HSCs). Studies utilizing *HBO1* gene-deficient mice have revealed that HBO1 deficiency leads to pancytopenia in both blood and bone marrow within 2–6 weeks of *HBO1* gene deletion, ultimately resulting in death due to hematopoietic failure. HBO1-deficient mice exhibit significantly reduced numbers of hematopoietic stem cells and progenitor cells, as well as diminished levels of peripheral blood cells, lymphocytes, and monocytes. HSCs in vivo can undergo three types of division: symmetric self-renewal, asymmetric division producing one HSC and one multipotent progenitor cell, and symmetric differentiation yielding two multipotent progenitor cells [80, 81]. Interestingly, HBO1 deficiency disrupts symmetrical self-renewal of HSCs, with all divisions potentially leading to symmetrical differentiation. Competitive transplantation experiments further demonstrate the involvement of HBO1 in maintaining the repopulating ability of HSCs, underscoring its critical role in HSC pool stability. Genome analysis of *HBO1* knockout mice has revealed downregulation of genes crucial for HSC function, including *Hoxa9*, *Pbx1*, *GATA2*, *Mpl*, *Itga2b*, and *Irf8*. These genes play pivotal roles in HSC quiescence, proliferation, and development [82, 83]. For instance, *Mpl* is involved in HSC quiescence and proliferation, while *GATA2* is essential for hematopoietic stem/progenitor cell (HSPC) development [84, 85]. Moreover, HOX proteins and their cofactors, such as Pbx1 and Meis1, are critical for cell identity during embryonic development and adult hematopoiesis, with *Hoxa9* deficiency resulting in reduced long-term repopulating ability of HSCs [86, 87]. Notably, the expression of genes essential for stem cell function is dependent on elevated levels of H3K14ac at their respective loci [88]. HBO1 deficiency leads to decreased H3K14ac levels, particularly at genes crucial for hematopoietic stem cell function. This suggests that HBO1 promotes the expression of a transcription factor network through its histone acetyltransferase (HAT) activity, which is indispensable for the maintenance and self-renewal of HSCs during adult hematopoiesis [89].

HBO1 is also involved in the normal differentiation of neural stem cells. Neural stem cells in the forebrain possess the remarkable ability to differentiate into neurons, astrocytes, and oligodendrocytes [90, 91]. HBO1 has been identified as a critical factor required for the differentiation of neural stem/progenitor cells (NSPCs). Studies utilizing mouse NSPCs lacking HBO1 have revealed that these cells exhibit slow proliferation rates for at least 15 generations and are unable to differentiate into neurons and oligodendrocytes, but can only differentiate into astrocytes. Deletion of HBO1 results in a decrease in the level of H3K14ac in cells, accompanied by the downregulation of more than 1000 genes. Interestingly, these downregulated genes are not necessary for NSPC proliferation in vitro but are crucial for nervous system development, neuronal differentiation, synaptic assembly, and behavioral regulation. Additionally, genes that are normally upregulated during normal differentiation are not activated in HBO1 knockout cells. These genes are known to play specific roles in neuronal differentiation, including axon guidance, neuroactive ligand–receptor interaction, synaptic function, and cognition. For instance, SOX2, a transcription factor proposed to initiate neuronal processes by activating genes such as *Neurod1* [92], requires HBO1 for its activation of target genes during differentiation. The absence of HBO1 during the middle stage of neurogenesis leads to abnormal cortical development and increased cell death. HBO1 knockout mice exhibit reduced cerebral cortex depth, increased cell density, enlarged lateral ventricles, smaller corpus callosum diameter, underdeveloped hippocampal structure, and underdeveloped dentate gyrus compared with control mice. Interestingly, reexpression of HBO1 after a short duration of deletion rapidly restores NSPC differentiation potential. However, delayed reexpression only partially restores NSPC plasticity, requiring long-term reexpression for full restoration [93]. In conclusion, HBO1-mediated H3K14ac plays pivotal roles in the normal differentiation and brain development of NSPCs, highlighting their importance in neurogenesis and brain function.

## HBO1 is closely related to many diseases

The expression of HBO1 has been closely associated with the development of various diseases. In several primary human tumor types, including testicular cancer, ovarian cancer, breast cancer, gastric/esophageal cancer, and bladder cancer, HBO1 protein expression was found to be strongly upregulated [11]. In non-small cell lung cancer (NSCLC), the transcription level of *HBO1* is increased, and *HBO1* silencing or knockout has been shown to strongly inhibit cancer cell viability, proliferation, and migration, while its ectopic overexpression enhances these processes. H3–H4 histone acetylation and the expression of several potential oncogenes (*CCR2*, *MYLK*, *VEGFR2*, and *OCIAD2*) were significantly reduced in NSCLC cells with *HBO1* silencing or knockout, suggesting that HBO1 may promote cancer cell growth through its HAT activity [94]. Similar patterns of HBO1 expression and function have been observed in osteosarcoma and liver cancer cells, indicating a potential role in promoting cancer development via similar mechanisms [95, 96]. Conversely, downregulation of HBO1 has been found to alleviate the activation of hepatic stellate cells, inhibiting liver fibrosis [97]. HBO1 has also been implicated in the activation of the Wnt/β-catenin signaling pathway, contributing to the development of human glioblastoma, B-cell acute lymphoblastic leukemia, and bladder cancer [30, 98, 99]. Interestingly, HBO1 expression is significantly reduced in bronchial epithelial cells (HBEC) of patients with chronic obstructive pulmonary disease (COPD). Experimental studies in emphysema model mice have demonstrated that HBO1 can mitigate HBEC apoptosis and emphysema induced by cigarette smoke extract (CSE), suggesting a protective role for HBO1 in COPD pathogenesis [100].

WM-3835 (*N*′-(4-fluoro-5-methyl-[1,1′-biphenyl]-3-carbonyl)-3-hydroxybenzenesulfonohydrazide), a specific HBO1 inhibitor, has been developed. WM-3835 can reduce the activity of acute myeloid leukemia tumor cells by inhibiting the level of H3K14Ac regulated by HBO1 and further reducing the transcription of *HOXA9* and *HOXA10* [31]. In addition, WM-3835 also targets HBO1 to inhibit the development of castration-resistant prostate cancer (CRPC) [101], NSCLC [94], osteosarcoma [95], and other tumors. We hope that the advent of some drugs based on WM-3835 will bring a new dawn to treatment of HBO1-related diseases by inhibiting HBO1.

## Summary and prospects

HBO1 exhibits a wide array of functions in cell biology, ranging from cell proliferation and gene expression to immune regulation, stem cell development, and cancer. However, several aspects of HBO1’s activity and regulation remain to be fully understood. One key area for investigation is the substrate selectivity of HBO1 acetyltransferase. Understanding which histone residues and nonhistone proteins are targeted by HBO1 will provide insights into its diverse cellular functions. Additionally, the specific mechanism by which the N-terminal domain regulates HBO1 activity requires further elucidation, as it likely plays a crucial role in modulating HBO1’s function. Moreover, HBO1’s involvement in DNA replication presents an intriguing area for exploration. Clarifying HBO1’s precise role in this process and its interaction with other replication factors will enhance our understanding of DNA replication regulation. Structural studies aimed at deciphering the complete structure of HBO1 are essential for comprehensively understanding its function. Such studies will shed light on the interaction between HBO1 domains, cofactor binding, and aid in the design of HBO1-targeting molecules for therapeutic purposes. The interaction between HBO1 and MEAF6, as well as MEAF6’s specific role within the HBO1 complex, remains poorly understood and warrants further investigation. Furthermore, the relationship between HBO1’s acetyltransferase activity and disease pathogenesis requires special attention, particularly in cancer where HBO1 is highly expressed. Elucidating the specific functions of HBO1 in cancer cells could uncover novel therapeutic targets for cancer treatment.

## Data Availability

Not applicable.
